# Anatomy features of the shoulder joint in asymptomatic chinese Han adults

**DOI:** 10.1186/s12891-023-06172-9

**Published:** 2023-01-28

**Authors:** Zheng Zeng, Miaomiao Liu, Yang Liu

**Affiliations:** Department of Orthopaedic Surgery, Beijing Tiantan Hospital, Capital Medical University, Beijing, China

**Keywords:** Shoulder pain, Scapula, Rotator cuff tears, Osteoarthritis, Prospective study

## Abstract

**Background:**

To evaluate the shoulder anatomical characteristics in asymptomatic Chinese adults.

**Methods:**

The prospective study enrolled individuals without shoulder pain at Beijing Tiantan Hospital Affiliated to Capital Medical University between January 2019 and January 2020. Six radiographic parameters were measured and analyzed, including glenoid plane to the acromion (GA), glenoid plane to the lateral aspect of the humeral head (GH), acromion index (AI), lateral acromial angle (LAA), acromion-humeral interval (AHI), and critical shoulder angle (CSA).

**Results:**

103 participants (51 males and 52 females) were enrolled. The mean values of GA, GH, AI, CSA, LAA, and AHI were 32.88 ± 5.68 mm, 47.16 ± 4.82 mm, 0.70 ± 0.11, 37.45 ± 6.00°, 6.32 ± 3.99°, and 9.611.86 mm, respectively. Females had lower GA (30.78 ± 5.06 vs. 35.01 ± 5.51 mm, *P* < 0.001) and GH (44.28 ± 3.67 vs. 50.11 ± 4.02 mm, *P* < 0.001) than males and LAA was significantly smaller in the Bigliani flat type compared with the curved type and the hooked type (5.07 ± 2.31° vs 12.33 ± 5.46°vs 10.00 ± 3.37, *P* = 0.001).

**Conclusions:**

Females had lower GA and GH than males in asymptomatic Chinese Han adults. Asymptomatic Chinese Han subjects with Bigliani flat type had lower LAA. CSA appears lager in Chinese Han individuals. Curve type of acromion performed lager LAA. The results may help establish an anatomical model of the shoulder joint and elucidate the anatomy features of the shoulder joint in asymptomatic Chinese Han adults.

## Background

Shoulder pain is the third most common musculoskeletal reason for primary care visits, after back and knee pain, and is reported to account for about 4–26% of all musculoskeletal complaints [1–3]. Shoulder pain primarily results from musculotendinous/ligamentous and joint-related conditions of the shoulder complex [4, 5]. Radiating pain may also be referred from the neck or due to irritation of the diaphragm. Pain may also be due to systemic conditions, infection, or tumors [1, 6]. Common shoulder presentations include acute shoulder injury due to contact sports or direct trauma, particularly from falls, bicycle accidents, motor vehicle collisions, similar high-impact trauma, and chronic atraumatic conditions such as tendinosis and arthritis [1, 7]. Indeed, chronic shoulder pathologies, including rotator cuff tears (RCT), subacromial impingement syndrome (SIS), and glenohumeral osteoarthritis (OA), are most likely a combination of functional factors, trauma, and degeneration [8].

Many previous studies focused on the anatomic variations of the acromion and its potential relation to chronic shoulder pathologies. For stratifying or quantifying the acromial variations, several parameters, including the acromial type [9], the acromial index (AI) [10], and the acromial tilt [11], have been reported. Recently, the critical shoulder angle (CSA) was reported by Moor et al. [12] and can quantify the extent of acromial coverage and the inclination of the glenoid. The CSA is defined by the angle between a line connecting the superior margin to the inferior margin of the glenoid and a line connecting the inferior margin of the glenoid to the inferolateral edge of the acromion.

Differences in CSA lead to significant changes in joint force vectors [13]. A high CSA involves a more lateral deltoid origin, leading to lesser compressive vectors of the deltoid on the glenohumeral joint [13]. A high CSA can result in altered vectors of the deltoid and stronger shear forces on the rotator cuff muscles, potentially leading to RCT and pain [14]. Compared with a CSA of 35°, a CSA of 38° requires 35% more force from the supraspinatus muscle to achieve shoulder stability [13]. A CSA > 35° is associated with RCT [12]. The scapula of the East Asian population is relatively smaller than in the North American population [8], leading to differences in shoulder joint mechanics. Nevertheless, whether CSA differs among ethnicities is still unclear.

Therefore, this study aimed to examine the shoulder joint anatomical factors [glenoid plane to the acromion (GA), glenoid plane to the lateral aspect of the humeral head (GH), AI, lateral acromial angle (LAA), acromion-humeral interval (AHI), and CSA] in asymptomatic Chinese Han adults. It’s possible that the existing shoulder joint parameters established for other populations may not correctly reflect the characteristics of the asymptomatic Han population, therefore reference values specifically to this population are needed in order to further understand the pathophysiology of RCT and OA of Han adults.

## Methods

### Study design and participants

This prospective study enrolled individuals without shoulder pain at Beijing Tiantan Hospital Affiliated to Capital Medical University between January 2019 and January 2020. This study was approved by the Ethics Committee of Beijing Tiantan Hospital Affiliated to Capital Medical University. All participants signed the informed consent.

The inclusion criteria were 1) 20–79 years of age, 2) normal shoulder movement and no shoulder pain in the past year, 3) physical examination as Neer test, Hawkins test and Jobe test were negative, and 3) clear consciousness and no cognitive impairment. The exclusion criteria were 1) history of shoulder joint disorders such as previous shoulder surgery, neuromuscular disease, RCT, SIS, trauma, inflammation, dislocation, rheumatoid arthritis, OA, etc., 2) medical history includes chronic wasting disease, such as a malignant tumor, pulmonary tuberculosis, cachexia, unstable vital signs, etc., 3) contraindications to X-ray, or 4) serious degree of motion artifact in X-ray.

### Procedures

All anteroposterior and lateral radiographs were taken in relaxed and comfortable standing positions using the same digital radiography equipment (Discovery XR656, GE Healthcare, Waukesha, WI, USA).. For anteroposterior radiographs, the subjects were placed in the seated position, and the side shoulder joint was closely pressed against the cassette. The upper limb was placed in a neutral position, and the center X-ray was tilted at 20°, and the radiation was from the glenohumeral joint. For lateral radiographs, the patient was placed in the sitting position, and the lateral upper limb adduction was in close contact with the cassette, with the central X-ray perpendicular to the medial edge of the scapula.

**Fig. 1:**
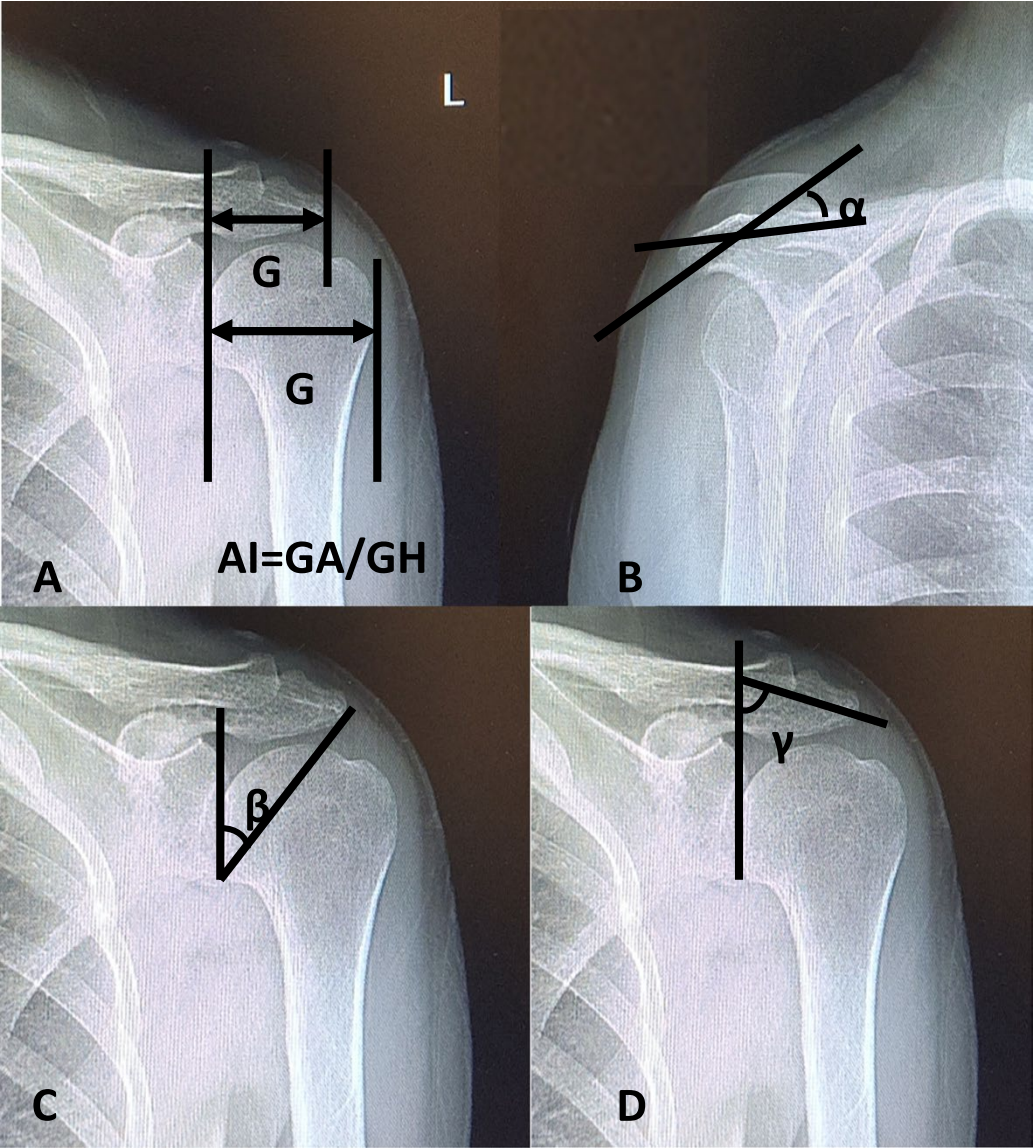
Radiographic parameters of shoulder joint parameters. **A** acromion index, **B** acromion slope, **C** critical shoulder angle, **D** lateral acromial angle

Six widely used representative radiographic parameters were measured in the Neusoft PACS software [15–17], including GA, GH, AI, CSA, LAA, and AHI **(**Table 1**) (**Fig. 1**)**. The radiographs were imported into the Mimics 14.0 software (Materialise, Leuven, Belgium) to obtain the 3D reconstruction and establish three-dimensional models of the bilateral shoulder joint. The DICOM format scan data was converted to the STL format file. After importing the 3D model into the MarkerBot printing software in STL format, a 1:1 physical model was printed to establish the “anatomical model of shoulder joint”. All data were blindly assessed by two orthopedic surgeons three times.

**Table 1:** Measurement methods for shoulder parameters

Shoulder parameters	Measurement
GA	The distance from the glenoid plane to the acromion.
GH	The distance from the glenoid plane to the lateral aspect of the humeral head.
Acromion index	The distance from the glenoid plane to the acromion [GA]/the distance from the glenoid plane to the lateral aspect of the humeral head [GH].
Acromial slope	The angle between the lines following the anterior 1/3 lower surface and posterior 2/3 lower surface of the acromion.
Critical shoulder angle	Measured between a line connecting the inferior with the superior border of the glenoid fossa and another connecting the inferior border of the glenoid with the most inferolateral point of the acromion.
Lateral acromial angle	One line was drawn along the superior- and inferior-most lateral points of the glenoid and represented the glenoid surface. Another line was drawn parallel to the acromion undersurface. The angle between these two lines.

The general data the participants were exacted from their medical records. The general characteristics included age, sex, dominant side of shoulder joint, and Biglianni type. Based on Bigliani classification, the acromion morphology is divided into type I (flat),type II (curved) and type III (hooked).

### Statistical analysis

The data were analyzed using SPSS 20.0 (IBM, Armonk, NY, USA). All continuous data were tested for normal distribution using the Kolmogorov-Smirnov test. Continuous data were presented as means ± standard deviation and analyzed using Student’s t-test. Categorical variables were presented as n (%) and analyzed using the chi-square test. The correlations between the variables were evaluated using the Pearson and Spearman correlation coefficient (Spearman for rank variable). *P*-values < 0.05 were considered statistically significant.

## Results

Each subject performed three trials. Shoulder joint parameters were measured by a joint surgeon, an orthopedic surgeon and a radiologist respectively in a double-blind manner. The intra-class correlation coefficients (ICC) ranged from 0.91 to 0.99.

103 participants (51 males and 52 females) were enrolled in this study. The mean age was 51.3 ± 12.8 years. 31 participants were left shoulder dominant and 72 right side dominant. According to the Bigliani classification, there are 84 flat, 15 curved, and 4 hooked. In all participants, the values of GA, GH, AI, CSA, LAA, and AHI were 32.88 ± 5.68 mm, 47.16 ± 4.82 mm, 0.70 ± 0.11, 37.45 ± 6.00°, 6.32 ± 3.99°, and 9.611.86 mm, respectively. **(**Table 2**)**. Females had lower GA (30.78 ± 5.06 vs. 35.01 ± 5.51 mm, *P* < 0.001) and GH (44.28 ± 3.67 vs. 50.11 ± 4.02 mm, *P* < 0.001) than males and LAA was significantly smaller in the Bigliani flat type compared with the curved type and the hooked type (5.07 ± 2.31° vs 12.33 ± 5.46°vs 10.00 ± 3.37, *P* = 0.001). Otherparameters showed no significant differences in the different categories of participants. There were no statistically correlation between age and shoulder imaging parameters (Table 3). ANOVA analysis showed LAA was significantly different among different Bigliani types (Table 4).

**Table 2:** Characteristics of the participants and shoulder parameters

Variables	n (%)	GA (mm)	GH (mm)	AI	CSA°	LAA°	AHI (mm)
Total	103	32.88 ± 5.68	47.16 ± 4.82	0.70 ± 0.11	37.45 ± 6.00	6.32 ± 3.99	9.611.86
Sex							
Male	51 (49.5)	35.01 ± 5.51^a^	50.11 ± 4.02^a^	0.70 ± 0.10	35.75 ± 5.60	6.12 ± 3.83	9.67 ± 1.47
Female	52 (50.5)	30.78 ± 5.06	44.28 ± 3.67	0.70 ± 0.12	38.13 ± 6.35	6.52 ± 4.16	9.55 ± 2.21
Age (years)							
< 30	4 (3.1)	34.05 ± 4.81	49.01 ± 2.78	0.69 ± 0.08	36.75 ± 2.95	4.75 ± 2.77	11.38 ± 1.33
30–39	19 (18.4)	33.36 ± 6.87	47.5 ± 4.64	0.7 ± 0.13	39.33 ± 8.73	5.5 ± 2.27	9.57 ± 2.43
40–49	20 (19.5)	33.96 ± 5.72	47.64 ± 5	0.71 ± 0.09	38.11 ± 5.07	7.05 ± 4.43	9.57 ± 1.8
50–59	34 (33.0)	32.37 ± 4.67	47.26 ± 3.82	0.69 ± 0.09	36.85 ± 5.27	5.67 ± 3.58	9.66 ± 1.29
60–69	24 (23.3)	31.07 ± 5.55	45.28 ± 5.64	0.7 ± 0.12	36.42 ± 5.12	7.92 ± 4.72	9.49 ± 2.13
> 70	5 (4.9)	36.88 ± 4	51.06 ± 3.25	0.72 ± 0.05	37.6 ± 4.92	4.4 ± 2.87	9.05 ± 1.2
Detection dominant side							
Left	31 (30.1)	32.9 ± 4.8	47.33 ± 4.62	0.7 ± 0.09	36.81 ± 5.2	7.32 ± 5.53	9.88 ± 2.57
Right	72 (60.9)	32.81 ± 6	47.09 ± 4.87	0.7 ± 0.11	37.72 ± 6.26	5.89 ± 2.96	9.51 ± 1.45
Bigliani type							
Flat	83 (80.6)	33.16 ± 5.53	47.37 ± 4.75	0.7 ± 0.11	37.42 ± 6.13	5.07 ± 2.29	9.73 ± 1.9
Curved	15 (14.6)	30.6 ± 5.94	45.28 ± 4.26	0.67 ± 0.12	37.67 ± 5.65	12.33 ± 5.27	8.92 ± 1.59
Hooked	5 (4.9)	34.5 ± 5.23	49.89 ± 5.53	0.69 ± 0.06	37.25 ± 2.95	10 ± 2.92	10.17 ± 1.57

^a^represent significant difference. *GA* Glenoid plane to the acromion, *GH* Glenoid plane to the lateral aspect of the humeral head, *AI* Acromion index, *CSA* Critical shoulder angle, *LAA* Lateral acromial angle, *AHI* Acromion-humeral interval

**Table 3:** Correlation analysis of age, Bigliani type and imaging parameters

	GA (mm)		GH (mm)		AI		CSA°		LAA°		AHI (mm)	
	r	P	r	P	r	P	r	P	r	P	r	P
Age ^(a)^	−0.154	0.120	−0.098	0.324	−0.058	0.559	−0.172	0.082	0.027	0.786	−0.103	0.299
Bigliani type ^(b)^	−0.119	0.232	−0.071	0.474	−0.096	0.337	0.023	0.818	.549**	< 0.000	−0.131	0.187

(a) Correlation between the parameter was performed by pearson correlation analysis (age and radiographs parameters were continuous variables). (b) Correlation between the parameter was performed by Spearman correlation analysis (Bigliani classification were classified variables). *GA* Glenoid plane to the acromion, *GH* Glenoid plane to the lateral aspect of the humeral head, *AI* Acromion index, *CSA* Critical shoulder angle, *LAA* Lateral acromial angle, *AHI* Acromion-humeral interval. *, *P*<0.05; *, *P*<0.01

**Table 4:** Correlation analysis between Bigliani type and LAA

Bigliani type(I)	Bigliani type(J)	Mean (I-J)	Standard error	*P* value
Flat type	Curve type*	−7.26**	0.84	<0.001
	Hook type*	−4.93*	1.53	0.005

*LAA* Lateral acromial angle. *p<0.05

## Discussion

The results suggest that females had lower GA and GH than males in asymptomatic Chinese Han adults. Compared with the literature, CSA appears to be larger in Chinese Han than in individuals from Japan, Brazil, and Switzerland. In addition, the results may help establish an anatomical model of the shoulder joint and elucidate the anatomy features of the shoulder joint in asymptomatic Chinese Han adults.

In the present study, 103 asymptomatic Chinese Han adults were enrolled, and shoulder parameters were obtained. The CSA values obtained from this study are similar to a previous study done on Chinese Han adults [18]. The CSA was larger in Chinese Han individuals than in individuals from Japan [19], Brazil [20], and Switzerland [12]. Surprisingly, although Chinese and Japanese are both East Asian populations, significant differences in CSA were observed. Low CSA may be a national specificity for Japanese Yamato adults because Japan is almost a monoethnicity country. Since CSA is a prognostic factor for tendon re-rupture after rotator cuff injury [21], Chinese Han adults will have a relatively high rate of postoperative complications.

Previous studies showed that shoulder pain represents 4–26% of all musculoskeletal complaints [1–3]. The prevalence of RCT in the general population is 15–32% [22]. Vellingrini et al. [14] showed that a high CSA increases force vectors on the rotator cuff muscles, leading to pain and RCT. Moor et al. [12, 23] showed that a CSA > 35° is associated with RCT. In the present study, CSA was not associated with age, sex, or Bigliani type, which was also observed by Suter et al. [24]. Still, the mean CSA was > 35°, suggesting a high risk of developing RCT, although it is controversial [25]. Nevertheless, whether the prevalence of RCT in China is higher than in other countries is currently unknown. Sex differences were observed in GA and GH, which were observed in previous studies [15, 26], while other parameters were comparable between males and females. LAA was significantly smaller in the Bigliani flat type than the hooked type, but the results in the literature are inconsistent [27, 28].

The pathogenesis, diagnosis, and treatment of degenerative diseases of the shoulder joint could be illuminated by the research of anatomy and morphology parameters of the shoulder joint. The disease could be diagnosed and managed in the early stage. Early intervention could be started before developing chronic shoulder diseases, improving the clinical outcomes. A standardized classification for the instability of the shoulder joint has not been established [16, 17]. Therefore, the management of patients with shoulder joint instability mainly relies on the personal experience of the doctors. Watson et al. [29–31] reported that the shoulder joint instability might be associated with anatomical factors. Analyzing the anatomy parameters of shoulder joint instability might provide new methods for the surgical treatment of the disease.

This study has several limitations. Although the participants were prospectively enrolled, only individuals who reported themselves as being asymptomatic were included, and no actual patient with shoulder pain was included. The age range of the subjects is large, and the sample size should be expanded in the future study. All participants were from a single hospital, and the sample size was small. Range of motion and muscle strength, which can be involved in shoulder stability, were not tested.

## Conclusions

In conclusion, females had lower GA and GH than males in asymptomatic Chinese Han adults. CSA appears lager in Chinese Han individuals. Curve type of acromion performed greater LAA than hook type and flat type. The results may help establish an anatomical model of the shoulder joint and elucidate the anatomy features of the shoulder joint in asymptomatic Chinese Han adults.

## Data Availability

All data generated or analysed during this study are included in this published article.

## Abbreviations

GA: Glenoid plane to the acromion
GH: Glenoid plane to the lateral aspect of the humeral head
AI: Acromion index
LAA: Lateral acromial angle
AHI: Acromion-humeral interval
CSA: Critical shoulder angle
RCT: Rotator cuff tears
SIS: Subacromial impingement syndrome
OA: Osteoarthritis
